# Structural Analysis of Glutaredoxin Domain of *Mus musculus* Thioredoxin Glutathione Reductase

**DOI:** 10.1371/journal.pone.0052914

**Published:** 2012-12-26

**Authors:** Olena Dobrovolska, Elena Shumilina, Vadim N. Gladyshev, Alexander Dikiy

**Affiliations:** 1 Department of Biotechnology, Norwegian University of Science and Technology, Trondheim, Norway; 2 Genetics Division, Department of Medicine, Brigham and Women's Hospital and Harvard Medical School, Boston, Massachusetts, United States of America; George Washington University, United States of America

## Abstract

Thioredoxin glutathione reductase (TGR) is a member of the mammalian thioredoxin reductase family that has a monothiol glutaredoxin (Grx) domain attached to the thioredoxin reductase module. Here, we report a structure of the Grx domain of mouse TGR, determined through high resolution NMR spectroscopy to the final backbone RMSD value of 0.48±0.10 Å. The structure represents a sandwich-like molecule composed of a four stranded β-sheet flanked by five α–helixes, with the CxxS active motif located on the catalytic loop. We structurally characterized the glutathione-binding site in the protein and describe sequence and structural relationships of the domain with glutaredoxins. The structure illuminates a key functional center that evolved in mammalian TGRs to act in thiol-disulfide reactions. Our study allows us to hypothesize that Cys105 might be functionally relevant for TGR catalysis. In addition, the data suggest that the N-terminus of Grx acts as a possible regulatory signal also protecting the protein active site from unwanted interactions in cellular cytosol.

## Introduction

Thioredoxin (Trx) and glutaredoxin (Grx) systems are two major thiol pathways that control cellular redox homeostasis [1]. The Trx system is composed of thioredoxin reductase (TR), thioredoxin (Trx) and Trx peroxidase, whereas the Grx system consists of glutathione reductase (GR), glutathione (γ-Glu-Cys-Gly tripeptide; GSH), glutaredoxin (Grx) and glutathione peroxidase (GPx). In these systems, electron flow is directed from NADPH through GR and TR towards their respective protein substrates. GR and TR belong to the pyridine nucleotide-disulfide oxidoreductase family. They are homodimers and contain a tightly bound FAD molecule in each subunit [1]. Mammalian TR and GR were found to be structurally and functionally similar, although TR has an additional C-terminal selenocysteine-containing active site, which serves as a substrate for the N-terminal active site [2], [3], [4], [5], [6], [7].

Three TRs genes have been identified in humans, including TXNRD1 (cytosolic TR, TR1), TXNRD2 (mitochondrial TR, TR3) and TXNRD3 (thioredoxin glutathione reductase, TGR) [8], [9], [10], [11], [12], [13]. TGR is unusual among TRs in that it has an additional N-terminal Grx domain, which is fused to a canonical TR module [13], [14], [15], [16]. The amino acid sequence of the TR module of TGR is more closely related to TR1 than to TR3 [13] and its Grx domain has a monothiol CPHS catalytic motif [13], [14], [15], [17]. The active site motif of the Grx domain of TGR can receive electrons from either the TR module or from GSH, and the protein was proposed to function predominantly in disulfide bond formation and isomerization in sperm proteins during spermatogenesis [14]. Mammalian TGR exhibits broad substrate specificity and can reduce various components of both Trx and Grx systems [16]. In particular, it was demonstrated that TGR can catalyze reactions associated with Grx (deglutathionylation), GR (NADPH-dependent reduction of GSSG) and TR (NADPH-dependent reduction of Trx) activities. It was argued that Grx and GR activities of TGR are mediated by its Grx domain [16].

Structural characterization of proteins is an essential step for establishment of their functional peculiarities. Structures of platyhelminth TGR (pdb code 2V6O) and the Grx domain of human TGR (pdb code 3H8Q) have been recently determined. We previously reported NMR resonance assignments of full-length and shortened (lacking 22 N-terminal amino acids) forms of the Grx domain of *Mus musculus* TGR [18]. In the present work, we report solution structure of this Grx domain using high-resolution NMR spectroscopy. This Trx-fold structure validates the model of the Grx domain [19] and is consistent with the structures of other Grx. We further used the structure to carry out comparative sequence, structure and charge distribution analyses of Grx and Grx domains in order to explain structural and functional peculiarities of the TGR's Grx domain.

## Materials and Methods

Reduced glutathione (GSH) was purchased from Acros Organics, and oxidized glutathione (GSSG) from Sigma Aldrich. Both compounds were dissolved in a buffer containing 10 mM sodium phosphate, 10 mM NaCl, pH 7.5.

### Sample preparation

Protein expression and purification of a uniformly isotope labeled (^15^N/^13^C) His-tagged version of the full-length and shortened forms of the Grx domain of mouse TGR (hereafter Grx and sGrx, respectively) was carried out as described previously [18]. NMR samples of reduced 1 mM Grx or sGrx in 10 mM sodium phosphate buffer, 10 mM NaCl, 10 mM β-mercaptoethanol, in 95% H_2_O/5% D_2_O and 100% D_2_O, pH 7.5, were analyzed by NMR.

### NMR spectroscopy

NMR spectra were recorded at 298 K on a Bruker Avance 600 MHz spectrometer equipped with a 5-mm z-gradient TXI (H/C/N) cryoprobe. Three-dimensional ^13^C- and ^15^N-edited ^1^H Nuclear Overhauser Effect Spectroscopy (NOESY) spectra were recorded in D_2_O and H_2_O, respectively. NMR data were processed using Bruker XWinNMR, version 3.5. NMR spectral analysis was performed using CARA version 1.8.4.2.

### Structure calculation

NOE cross-peaks were identified, assigned and integrated in the aforementioned NOESY spectra using the CARA program. The CALIBA subroutine in CYANA 2.1 was used to convert cross peak intensities to distance constraints. Dihedral angle constraints were derived from secondary chemical shifts using the TALOS program [20]. Based on the input, the structure was calculated using the torsion angle dynamics program CYANA2.1 [21]. Twenty conformers with the lowest final CYANA target function values were further energy minimized in vacuum using AMBER force field with the aid of AMBER 9 program [22]. The mean structure was generated using MOLMOL 2k.2.0 [23] and further energy minimized in AMBER.

### Structure analysis

Quality of structures was analyzed using MOLMOL and PROCHECK - NMR [24]. The relevant figures and electrostatic potentials were prepared using MOLMOL version 2k.2.0.

### NMR experiments

In order to characterize the glutathione-binding site of Grx NMR ^15^N-^1^H HSQC titration experiments were performed. A 1 mM sample of ^15^N-labeled Grx in buffer, containing 10 mM sodium phosphate, 10 mM NaCl, pH 7.5, was titrated with unlabeled GSH and GSSG at room temperature in the following proportions: 1∶1/3; 1∶1/2; 1∶2/3; 1∶1; 1∶2; 1∶10, either in the absence or presence of 10 mM β-mercaptoethanol. In the membrane environmental modeling experiment, 5% w/v SDS was added to the solution of ^15^N-labeled Grx and the ^15^N-^1^H HSQC spectra of the mixture were recorded at 30°C and 42°C. For water exchange experiments, samples of ^15^N-labeled Grx or sGrx in the NMR buffer were lyophilized and further dissolved in D_2_O. A course of subsequent ^15^N-^1^H HSQC spectra for each protein was recorded every 30 min.

### Bioinformatics analysis

Protein multiple sequence alignments were performed with ClustalW [25]. Sequence similarity analysis was performed by the SIAS server (http://imed.med.ucm.es/Tools/sias.html). Structural superimposition was carried out using SuperPose [26]. An analysis of the N-terminal region (residues 1–22) was performed using iPSORT [27] and MITOPROT [28].

## Results and Discussion

### Structure description

**Figure 1:**
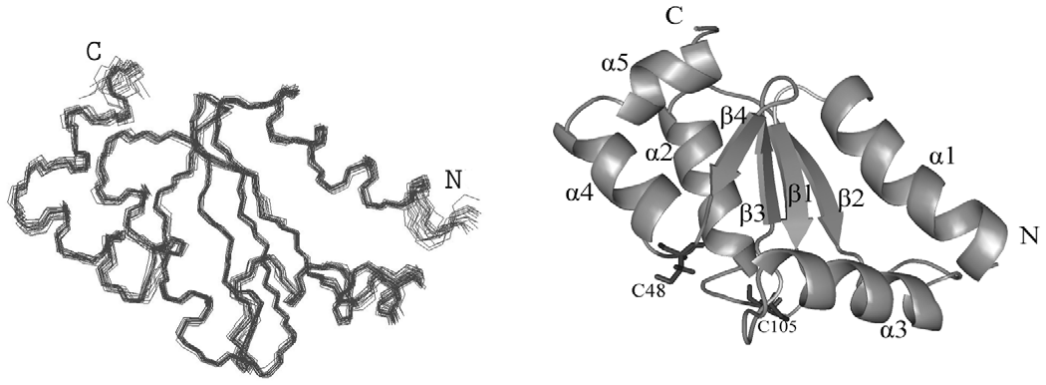
Solution structure of the reduced Grx domain of mouse TGR. Left: overview of backbone superimposition of 20 conformers with the lowest target function. Right: overview of the ribbon representation of the minimized conformer closest to the mean structure. The figure also shows the active site cysteine (C48) and C-terminal C105.

Solution structure of the Grx domain of mouse TGR was calculated based on the NOE-derived geometrical constraints and dihedral angles obtained from TALOS. The geometrical constraints used in the calculations are summarized in Table 1. In total, 894 NOE-based upper distance limits and 182 ψ and φ torsion angle restraints were used to derive the Grx structure. The resulting Grx family was further energy-minimized. The geometrical constraints and coordinate files of the minimized Grx family were deposited in the PDB under the code 2lv3. Figure 1 shows a superimposition of the final 20 minimized conformers with the lowest target function, together with a ribbon representation of the minimized conformer closest to the mean structure showing the secondary structure elements, active site cysteine C48, and C-terminal C105 (further discussed). The calculated structure is of high quality and fully corresponds to the experimentally determined constraints.

**Table 1:** Structural statistics and geometrical constraints derived from NMR for the reduced form of the Grx domain of mouse TGR.

Restraints used in structure calculation	Number
Total number of NOE distance restraints	894
Intrarresidual	161
Medium range	473
Long range	260
Torsion angle constraints	182
**Structure statistics, 20 conformers**	
CYANA target function value (Å^2^)	6.48±0.27
Maximal distance constraint violation (Å^2^)	0.44±0.18
Maximal torsion angle constraint violation (Å^2^)	22.79±0.9
AMBER energies in vacuum (kcal/mol)	−2.95E+3
**PROCHECK – NMR Ramachandran statistics**	
Residues in favourable regions (%)	87,8
Residues in additional allowed regions (%)	6,7
Residues in generously allowed regions (%)	3,3
Residues in disallowed regions (%)	2,2
**Root mean square deviation to average coordinates (Å)**	
N, C^α^, C^'^ (23–124)	0,48±0,10
Heavy atoms (23–124)	0.99±0,11

The N-terminal region of Grx (first 22 amino acids) was excluded from the structure calculation as most of the corresponding HSQC and NOE signals were not detected [18]; hence, the structure of Grx starts with Ala 23. Analysis of the structure shows that the Grx domain is a compact Trx-like spherical molecule with a central core of four-stranded β-sheets flanked on either side by five α-helices arranged in the order α_1_-β_1_-α_2_-β_2_-α_3_-β_3_-β_4_-α_4_-α_5_ (Figure 1). The N-terminal region begins with an α_1_ (residues Arg 24 - Glu 36), followed by β_1_ consisting of residues Val 40 to Ser 44. The active site Cys 48 - Ser 51 (-CPHS- motif) is situated on the unstructured loop between β_1_ and α_2_ (residues Arg 53–Ser 59). The strand β_2_ comprises residues Asn 66 to Glu 69; following a loop, α_3_ consists of residues Gly 76 to Ser 87, followed by β_3_ (Asn 94–Val 97) and β_4_ (Val 100–Gly 103). The C-terminal region includes α_4_ (residues Arg 107–Asn 114) and α_5_ (residues Leu 116–Leu 120), connected through a hinge section. Strands β_1_ and β_2_ are parallel, and strand β_3_ is antiparallel with β_1_ and β_4_. Helices α_1_ and α_3_ pack on one side of the β-sheet, whereas α_2_, α_4_ and α_5_ are on the other. Packing of the sandwich-like architecture is mainly maintained by hydrophobic interactions between the sheet and helices. The determined Grx structure shares significant structural similarity with the modelled Grx domain of mouse TGR [19].

### N-terminal region

**Figure 2:**
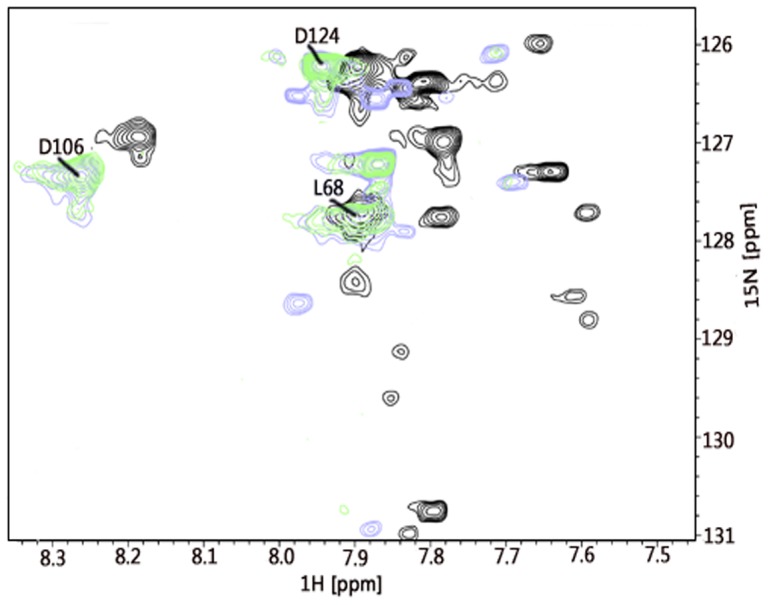
A fragment of ^15^N-^1^H HSQC spectra of the reduced Grx domain of mouse TGR. Green shows Grx HSQC spectrum at 30°C, light blue shows Grx HSQC spectrum in the presence of SDS at 30°C, and black corresponds to Grx HSQC spectrum in the presence of SDS at 42°C. For more details see the text.

As mentioned above, the N-terminal region of Grx was not detectable in HSQC and NOE spectra. The absence of the corresponding cross-peaks could be attributed to the higher mobility of this protein region. A decrease in temperature may slow down protein mobility and thus allow detecting the missing cross-peaks. Nevertheless, the ^15^N-^1^H HSQC spectra of Grx recorded at a lower temperature (8°C) did not show additional signals in the spectra (data not shown). Further analysis of the first 22 amino acids of Grx using iPSORT suggested the presence of a candidate mitochondrial targeting peptide. Indeed, the N-terminal sequence is rich in positively charged and hydrophobic residues that may constitute the targeting helix [29]. Analysis of the N-terminus performed by MITOPROT predicted the cleavage site after the first 19 residues (MSSPPGRRARLASPGTSRP). However, analysis of cellular distribution of TGR suggested that the enzyme occurs in the cytosol of spermatids at the time of mitochondrial sheath formation [14]. In these spermatids, TGR is accumulated near the site of mitochondrial sheath assembly. It was shown that mammalian sperm is stabilized by disulfide bond (S-S) bridges cross-linking thiol-rich proteins present in the membranes of sperm mitochondria [30]. Thus, TGR, which combines the elements of Grx and Trx systems, might be involved in disulfide bond formation during spermatogenesis. The N-terminus can also act as a regulatory sequence. Showing little structural organization in solution, mitochondrial targeting sequences are predicted to form amphipathic α-helixes in the membrane or membrane-like environment. The amphipathic nature of these structures is thought to be important for their specific recognition by the protein import machinery [31]. A membrane-like media are prepared by self-association of surfactants in aqueous solutions, which are divided into two large groups: detergents (form micelles) and lipids (form bilayers) [32]. For preparation of micelles, which are widely used in NMR structural studies, negatively charged SDS detergent is often used [33]. In NMR spectroscopy, the formation of the protein's secondary structure results in the appearance of a set of well-dispersed HSQC cross-peaks. To further examine the N-terminal part of Grx, we recorded ^15^N-^1^H HSQC spectra of the SDS-treated Grx domain at 30°C and 42°C [34]. The obtained ^15^N-^1^H HSQC pattern was shifted towards the lower field of both dimensions; however, it did not change dramatically, and, as expected, contained additional well-dispersed cross-peaks, as shown in Figure 2. The number of new cross-peaks corresponded to (but did not exceed that of) the number of amino acids constituting the N-terminal segment, therefore, confirming that the positively charged N-terminus becomes structured in the negatively charged environment.

Although the ^1^H-^15^N HSQC patterns of Grx and sGrx resemble each other, indicating overall structural correspondence of the two protein forms, several differences between them were observed. First, the full-length protein had a higher stability than the shortened form. Second, the signals in ^15^N-^1^H HSQC, corresponding to residues C105 and D106, were not found in Grx, while they were present and assigned in sGrx [18]. Weak HSQC signals and any NOESY patterns were also found for residues D74 and A77 in the corresponding spectra of Grx [18]. These findings correlating with the presence/absence of the N-terminus further highlight structural differences between the two protein forms (see the following paragraphs).

### Electrostatic potential

**Figure 3:**
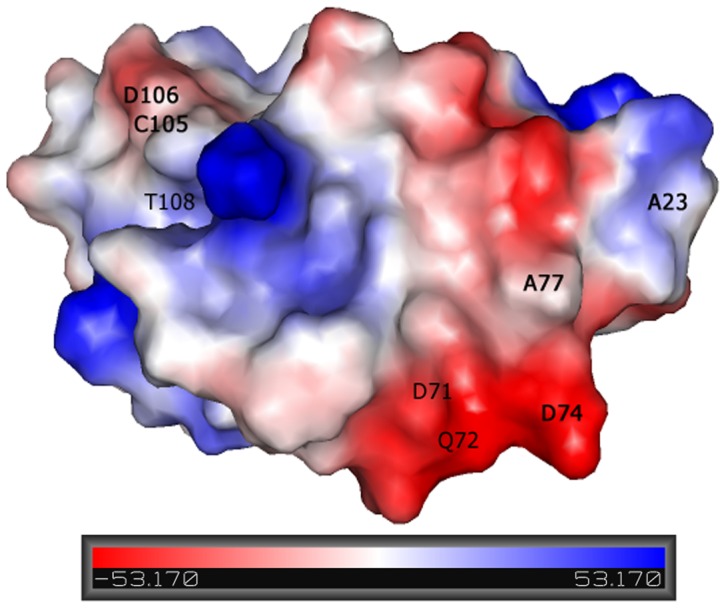
Surface charge distribution of the reduced Grx domain of mouse TGR.

Figure 3 shows electrostatic potential calculated for the obtained structure of Grx. It is apparent from the analysis of this figure, that the missing NOE signals of D74, A77, C105, D106 (see above) amino acids belong to the negatively charged region closest to the N-terminus. As the N-terminal region of Grx is composed of positively charged (R7, R8, R10, R18) and polar (S2, S3, S13, T16, S17, S20, S21) amino acids, their involvement in electrostatic interaction with negatively charged protein surface can be suggested. The broadening of the NMR signals corresponding to D74, A77, C105, D106 due to this interaction may be a reason that the mentioned amino acid signals in the NMR spectra of the full length form of Grx where not detected. Indeed, the resonances belonging to D74, A77, C105, D106, not observed in Grx protein, were firmly detected in sGrx lacking the mentioned electrostatic interaction. Interestingly, the active site motif of Grx (C48, P49, H50, S51), as monitored by our structural studies, resides on the neighbouring loop near residues C105 and D106. Thus, the N-terminal region, positioned in proximity to the active site, could shield it from the solvent and, therefore, protect from unwanted reactions stabilizing the full-length protein.

### D_2_O exchange experiments

To further comparatively characterize both Grx and sGrx proteins, we carried out water exchange experiments monitoring behaviour of ^15^N-^1^H HSQC spectra in D_2_O for both full-length and shortened forms of the ^15^N-labeled Grx domain of TGR. Interestingly, during the first 30 minutes in D_2_O, Grx exchanged ten residues more with respect to sGrx (D33, G37, N38, V40, S59, V63, D71, Q72, E85, T108). However, after 3 hours of incubation in D_2_O, both Grx and sGrx reveal an identical pattern of exchanged/not exchanged residues. Therefore, our experiments show that while the final rate of water exchange is the same both for Grx and sGrx, the short term dynamics of the water exchange is different for these proteins. The observed differences mostly regard residues belonging to the negative patch involved in the suggested interaction with the positively charged N-terminus (see above). The fact that these residues exchanged within the first 30 min in Grx, while in sGrx they exchanged only 3 hours later indicates that the N-terminus in some way promotes faster rates of water diffusion into Grx protein.

### Comparative sequence analysis of Grx

Grx occur in the majority of organisms in the three domains of life. Structures of many of these proteins were determined either by NMR or X-ray crystallography. Two main groups of Grx can be distinguished based on phylogeny, active site motifs, and domain structure: (i) ‘classical’ dithiol Grx containing the active site consensus sequence Cys-X-X-Cys (i.e., two Cys separated by two other amino acids); and (ii) monothiol Grx with a Cys-X-X-Ser active site consensus sequence. The latter Grx utilize only the N-terminal active site Cys in their catalytic mechanism, which is used together with two glutathione molecules, while dithiol Grx can use either one or both Cys in the active site. Both types of disulfides formed during Grx catalysis are reduced *in vitro* by GSH or TRs [35].

**Figure 4:**
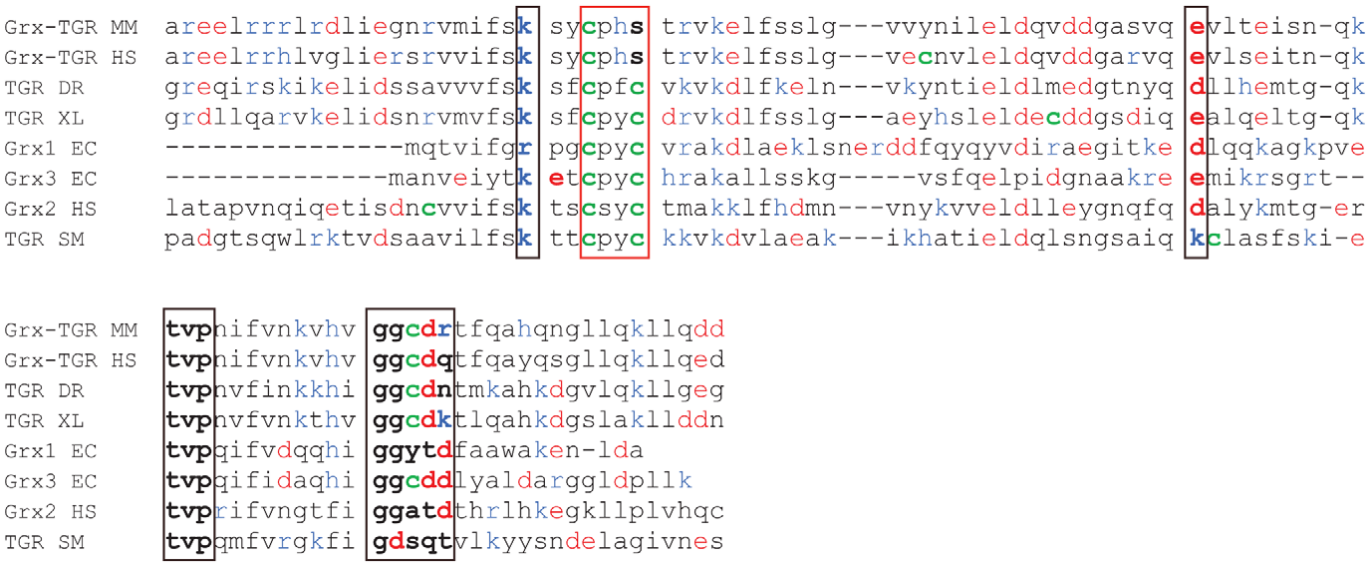
Amino acid sequence alignment of the Grx domain of mouse TGR with mono- and dithiol Grx from various organisms. The active site residues of Grx are highlighted with a red rectangle, and the residues involved in the interaction with glutathione are within black rectangles. Positive and negative charged amino acids are marked with blue and red colour, respectively. Cysteines residues are marked in green. Sequences abbreviation: HS, *Homo sapiens*; SM, *Schistosoma mansoni*; XL, *Xenopus laevis*; MM, *Mus musculus*; DR, *Danio rerio*; EC, *Escherichia coli*.

We analyzed an alignment of the Grx domain of mouse TGR with both Grx and Grx domains of TGR from various organisms, which contain mono- or dithiol active sites (Figure 4). The active site residues (highlighted with a red rectangle), and residues involved in the interaction with GSH (marked with black rectangles) are conserved in mono- and dithiol Grx, including the Grx of mouse TGR [36].

**Figure 5:**
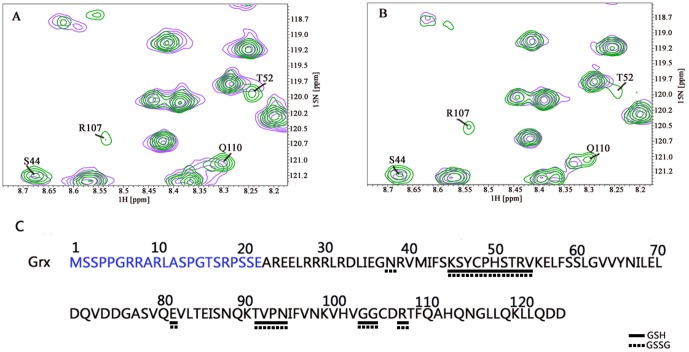
Fragments of ^15^N-^1^H HSQC spectra of ^15^N-labeled reduced Grx domain of mouse TGR titrated with unlabelled GSH and GSSG (panels A and B, respectively). Green corresponds to free Grx and magenta to Grx incubated with GSH/GSSG. Only the residues for which alteration of NMR parameters upon titration was observed are marked. Panel C: qualitative representation of the data. Solid and dashed horizontal lines below the Grx amino acid sequence highlight the residues interacting with GSH and GSSG, respectively.The N-term of Grx is marked in blue. For more details see the text.

Since interaction of the Grx domain with glutathione is assisted by electrostatic interactions, we next analysed the distribution of charged amino acids. As shown in the figure 4, the active site of Grx is surrounded by positively charged amino acids (marked in blue). Interestingly, only in *E. coli* Grx3 and the Grx domain of *Xenopus laevis* TGR, negative residues are found in the vicinity of the active site (Fig. 5, marked in red). It was suggested that these negative residues influence the redox potential of these proteins [37]. In addition, the C-terminal segment of the Grx domain of TGR from human, mouse, *Danio rerio*, *Xenopus laevis* and Grx3 from *E. coli* harbor an additional Cys residue. Our structural data indicate that the distance between the active site cysteine (Cys 48) and C-terminal Cys105 (Figure 1, right panel) is 10–15 Å. Although the distance might be too large for the formation of an intra-molecular disulfide bridge, according to our data there is no steric hindrance between these two cysteines located on unstructured loops. It can be hypothesised that the formation of an intra-molecular disulfide bridge between the two cysteine residues (e.g., as observed in mammalian MsrB1 protein [38], [39]) may have functional and, perhaps, even catalytic relevance.

### Structural comparison with other Grx

Our analysis, as well as other structural studies of a set of GSH-dependent Grx [26] revealed common elements present in their binding sites: i) a CXXC/S active site motif; ii) a Tyr or a Phe in close proximity to the catalytic Cys; iii) a TVP motif with Pro in the *cis* conformation; iv) a GG kink in proximity to the active site; and v) conservation of charged residues at both edges of the substrate binding groove (GSH binding pocket). The structure of the Grx domain of mouse TGR was generally similar to other Grx structures, but varied in secondary structure elements attached to a common Grx core. For example, an N-terminal α-helix (α_1_ in our structure) was present in many Grx including human TGR (PDB code 3H8Q), human Grx2 (PDB code 2FLS), Grx of *Sm*TGR (PDB code 2V6O), monothiol *E. coli* Grx4 (PDB code 1YKA), and poplar Grx C1 (PDB code 2E7P), but not in the dithiol *E. coli* Grx3 (PDB code 1FOV). A lack of C- and/or N-terminal α-helices together with the length of the loop preceding the active site motif, was suggested to play a key role in constraining the degree of conformational adaptability for substrate binding displayed by Grx [40]. Minor structural differences were also observed between the Grx domain of mouse TGR and the Grx domain of *Sm*TGR: the latter was characterized by a shorter C-terminal α-helix [41].

Recent studies [42] demonstrated that *Sm*TGR may function *via* two catalytic mechanisms: monothiol and dithiol. In the monothiol mechanism, when the GSH concentration is high, glutathionylated catalytic Cys (Cys28) of Grx gets resolved by GSH. At low GSH concentrations, a second, dithiol mechanism applies, wherein the C-terminal Cys (Cys31) in the CXXC motif acts as a resolving group, breaking the disulfide bond between Cys28 and GSH, forming an internal Cys28-Cys31 disulfide and releasing GSH. An oxidized Grx can be further reduced by the redox-active Cys/Sec–Cys pair of the TR domain [43]. The same study analyzed the deglutathionylation activities of a *Sm*TGR variant, in which Cys31 was replaced with Ser (making it analogous to mouse TGR), which exhibited 22% of wild type *Sm*TGR activity. This study suggested that the role of the second Cys in the monothiol mechanism is to stabilize the thiolate anion of the N-terminal Cys through a hydrogen bond, thus facilitating its nucleophilic attack on GSSG.

### Grx titration with GSH/GSSG

Figure 5 illustrates fragments of the ^15^N-^1^H HSQC titration of the Grx domain with reduced (Figure 5A) and oxidized (Figure 5B) glutathione. Upon interaction with these two molecules the chemical environment of the nuclei involved in the interaction changes, which results in perturbation of the corresponding NMR signal: i.e., chemical shift change or signal broadening occurs. By performing NMR titration of ^15^N-labeled Grx with its unlabelled partners, GSH and GSSG, we monitored their interaction and mapped the Grx residues involved in binding with the respective partners.

Upon titration of Grx with either GSH or GSSG, nearly 90% of the signals remained unaltered; however, some of the signals appear changed (mostly broadened). The titration experiments of the Grx domain titration with GSH revealed signal broadening that corresponded to the following amino acids: S44, K45, C48, P49, H50, S51, T52, R53, V54, E81, T91, V92, P93, N94, G103, G104, R107. Grx titration with GSSG showed changes for the following residues: N38, K45, C48, P49, H50, S51, T52, R53, V54, E81, T91, V92, P93, N94, G103, G104, and R107 (Figure 5C). Interestingly, amino acids observed in the two experiments almost coincided. These experimental data not only point to the glutathione binding site in the Grx domain of TGR, but also suggest that the binding sites for reduced and oxidized glutathione largely overlap.

## Conclusions

This study describes the NMR solution structure of the monothiol Grx domain of mouse TGR. As expected, the protein possesses a Trx fold and consists of a four-stranded β-sheet flanked by five α-helices. The active site motif containing the catalytic redox-active Cys is located on the protruding loop connecting strand β1 and α2.

Analysis of the N-terminal segment of Grx, which was not included in the structure determination, showed that it has features of a targeting sequence or a regulatory region. It was found, by analyzing ^15^N-^1^H HSQC spectra, that this segment becomes structured when protein is treated with a detergent, thereby mimicking membrane-like environment. Based on the analysis of surface charge distribution of the protein, we suggest that the N-terminus resides near the active site, shielding it from redox interactions.

Sequence alignment of the domains with other Grx and Grx domains revealed a characteristic GSH-binding site, which was further characterized with the help of NMR. The data suggest a significant overlap between the GSH and GSSG binding sites. Further analysis of mammalian TGR function would require structural information of the entire enzyme.
